# Case Report: X-linked agammaglobulinemia with progressive neurodegeneration from immunological to neurological implications

**DOI:** 10.3389/fimmu.2025.1611748

**Published:** 2025-08-29

**Authors:** Xiaoyi Chen, Hao Gu, Yurong Piao, Yanli Ma, Yiran Zhao, Huawei Mao, Yuan Wang, Jie Deng

**Affiliations:** ^1^ Department of Neurology, Children’s Hospital Affiliated to Zhengzhou University, Henan Children’s Hospital, Zhengzhou Children’s Hospital, Zhengzhou, China; ^2^ Department of Immunology, Beijing Children’s Hospital, Capital Medical University, National Center for Children’s Health, Beijing, China; ^3^ Department of Neurology, Beijing Children’s Hospital, Capital Medical University, National Center for Children’s Health, Beijing, China

**Keywords:** XLA, Btk gene, progressive neurodegeneration, chronic active infection, central nervous system

## Abstract

**Background:**

X-linked agammaglobulinemia (XLA) is a rare disorder associated with defective B-lymphocyte differentiation, also known as circulating B-cell deletion or deficiency, reduced levels of all serum immunoglobulin isoforms, and a lack of specific antibody production. XLA has rare neurological complications but has a refractory course and poor prognosis. Here, we report a case of XLA due to a Bruton tyrosine kinase gene variant with progressive neurodegeneration.

**Case description:**

We describe a boy with XLA who had recurrent infections since infancy and, after diagnosis was confirmed by genetic testing, was started on regular intravenous immunoglobulin at the age of 5 years. However, after a second episode of suspected meningitis at the age of 4.9 years, he developed motor and cognitive deterioration, becoming unable to sit, walk, eat or speak after 8 months, with frequent tremors and multiple seizures, and died of respiratory failure at the age of 7 years. Magnetic resonance imaging showed progressive whole brain atrophy. Combined with a mild lymphocytic inflammation of the cerebrospinal fluid, we suspected a chronic active infection of the central nervous system, but it was difficult to confirm our suspicion by serological testing due to the inability to produce neutralizing antibodies.

**Conclusions:**

Severe progressive neurodegeneration in XLA is rare. With this case we would like to discuss the difficulties in diagnosing infection in patients with XLA and the role of autoimmune mechanisms in the development of neurodegeneration.

## Introduction

X-linked agammaglobulinemia (XLA) is a congenital immunodeficiency disorder caused by mutations in the Bruton tyrosine kinase (*BTK*) gene (1), which encodes an essential protein required for the development and maturation of B cells. Alterations in the BTK gene impair B cell differentiation, resulting in a significant reduction in mature B lymphocytes, plasma cells, and all immunoglobulin (Ig) subtypes (2). As a result, individuals with XLA are highly prone to recurrent infections, particularly those affecting the respiratory tract due to encapsulated pyogenic bacteria. Additionally, gastrointestinal infections caused by pathogens such as Salmonella, Yersinia, Campylobacter, and Giardia are common. The primary treatment for XLA involves immunoglobulin replacement therapy, delivered through intravenous (IVIg), subcutaneous, or enzyme-mediated approaches (3, 4).

While XLA predominantly presents with recurrent infections, rare central nervous system (CNS) complications have been observed (5, 6). These include progressive neurodegenerative disorders and chronic neuroinflammations. Notably, these complications often occur without evidence of specific infectious agents, making diagnosis and treatment very difficult. To date, only a few cases have been documented, with limited information on the early clinical course of CNS involvement. In this report, we describe a case of XLA associated with progressive neurodegeneration and severe neurological symptoms. Importantly, this case provides a rare opportunity to follow the progression of CNS complications from onset to the patient’s ultimate outcome, providing important insights into the natural history of this rare complication in XLA.

## Case description

### Clinical course

**Figure 1:**
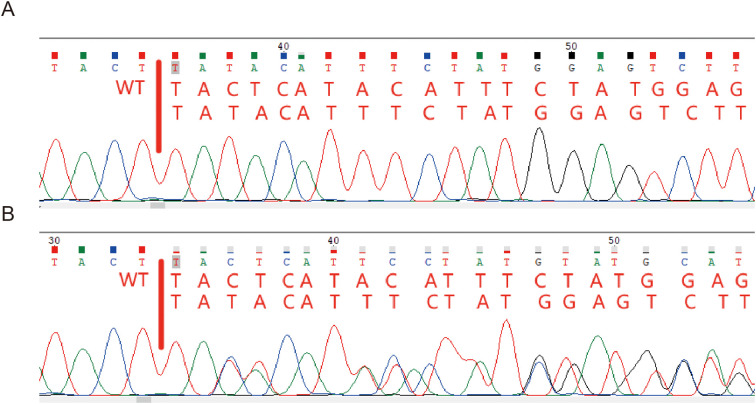
**(A)**. Sanger sequencing of the BTK gene (c.838_839 + 2del) of patient. **(B)**. Sanger sequencing of mother’s BTK gene.

A 6.7-year-old boy was admitted to our hospital for severe pneumonia and was treated with anti-infective therapy with ceftriaxone, meropenem and azithromycin. He experienced the first infection at the age of 2 months when he had pneumonia. Initial immune function testing at 3 months of age revealed a complete absence of B cells (0%), with markedly reduced serum immunoglobulin levels (IgG 0.7 g/L, IgA 0.01 g/L, IgM 0.4 g/L), suggesting an immunodeficiency disorder, but no further diagnostic workup was performed at that time. At the age of 3 years, he was diagnosed with septic meningitis in another hospital and received anti-infective treatment for 25 days, during which time he developed central expiratory failure and required endotracheal intubation. Meningitis was suspected a second time at the age of 4.9 years, and he received 21 days of anti-infective treatment, but no definitive pathogen was identified for either CNS infection. Repeat immunological testing at the age of 5 years confirmed the absence of mature B lymphocytes, with serum IgG levels of 4.59 g/L (after IVIg), IgA 0.01 g/L, and IgM 0.44 g/L, all below normal ranges. Genetic testing was then performed, and a maternal variant c.838_839 + 2del of the *BTK* was identified (**Figure 1**), confirming the diagnosis of XLA. In addition, he had recurrent otitis media, sinusitis, and pneumonia, and had been infected with pathogens including Haemophilus influenzae, Staphylococcus aureus, adenovirus, and SARS-CoV-2. He was subsequently started on regular IVIg replacement therapy at a dose of 400-500 mg/kg every 1-2 months. Despite achieving serum IgG levels within the reference range (6.47-19.56 g/L; normal range 6.3-15 g/L), he continued to experience frequent infections, averaging one episode per month, which were often prolonged and refractory to treatment.

Regarding past history, the boy is the only child of a nonconsanguineous healthy Chinese couple, delivered at 39 weeks of gestation, with a birth weight of 3.7 kg and an uneventful perinatal period. He had received BCG vaccine once and hepatitis B vaccine twice. Upon further verification of the vaccination history, we found that he had received the measles vaccine at 8 months of age. History of fever with rash was denied. There was no family history of related diseases, and the mother was the only child in the family. His early motor and intellectual developmental milestones were normal, and he could walk at 1 year of age and speak at 1 year and 2 months.

Following the second episode of suspected meningitis, the boy developed significant neurological complications, including hearing impairment, dysarthria and ataxic gait at the age of 5 years. Six months later, he experienced multiple afebrile seizures characterized by head shaking, limb twitching and loss of consciousness lasting more than 10 seconds each, which were treated with levetiracetam. Despite seizure control, his neurological function progressively deteriorated and within 8 months he lost the ability to turn over, sit independently, walk or speak. He also developed chewing and swallowing difficulties, requiring nasogastric feeding, and exhibited almost continuous postural tremors while awake. However, he rapidly became comatose and opisthotonos and required mechanical ventilation; repeated attempts to withdraw the ventilator were all failed. Unfortunately, the boy died of respiratory failure 2 months after this hospitalization at the age of 7 years.

### Laboratory findings

Flow cytometry analysis of T and B cell subsets revealed that the absolute count of T cells is normal. The proportions of T cell subsets, including CD4+ T cells and naive CD4+ T cells, were elevated, while the proportions and absolute counts of CD8+ T cell subsets at all stages were generally within the normal reference interval. Further examination of the T helper (Th) cell subpopulation revealed that the Th1, Th2, Th17 and Treg were all within normal reference ranges. Both the proportion and absolute count of B cells were undetectable, with no presence observed in any B cell developmental stage, including naive B cells, memory B cells, plasmablasts, and transitional B cells. In contrast, the proportion and count of natural killer (NK) cells were within the normal range (**Table 1**).

**Table 1:** Lymphocyte subsets of the patient.

			Reference	
	%	Abs #(/μl)	%	Abs #(/μl)
Lymphocytes				
T cells				
DNT	0.2	5	0.18-2.81	4-55
γδT	7.3	177	6.92-19.84	124-410
DPT	0	0	0.15-0.68	4-21
CD3+	85.6	2506	60.05-74.08	1424-2664
CD4+	45.7	1338	26.17-40.76	686-1358
CD4 CM	14.3	191	22.06-46.46	211-478
CD4 Naive	83.4	1116	45.56-75.28	321-972
CD4 EM	1.6	21	2.08-8.78	23-84
CD4 TEMRA	0.7	9	0.00-1.06	0-13
CD8+	33.7	986	19.68-34.06	518-1125
CD8 CM	13.1	129	12.08-30.54	85-268
CD8 Naive	59.1	583	41.58-77.90	297-730
CD8 EM	11.4	112	1.58-13.18	10-129
CD8 TEMRA	16.4	162	1.7-24.62	11-218
Th1	13.26		6.5-28	
Th2	1.31		0.3-2.2	
Th17	0.91		0.2-2.4	
Treg	6.59		4.1-9.4	
B cells				
B cell	0	0	10.21-20.12	280-623
Naive B	0	0	48.36-75.84	147-431
Memory B	0	0	7.76-19.90	31-94
Plasmablast	0	0	0.9-7.36	4-28
Transitional	0	0	2.58-12.30	10-66
NK cells	15.2	428	9.00-22.24	258-727

**Figure 2:**
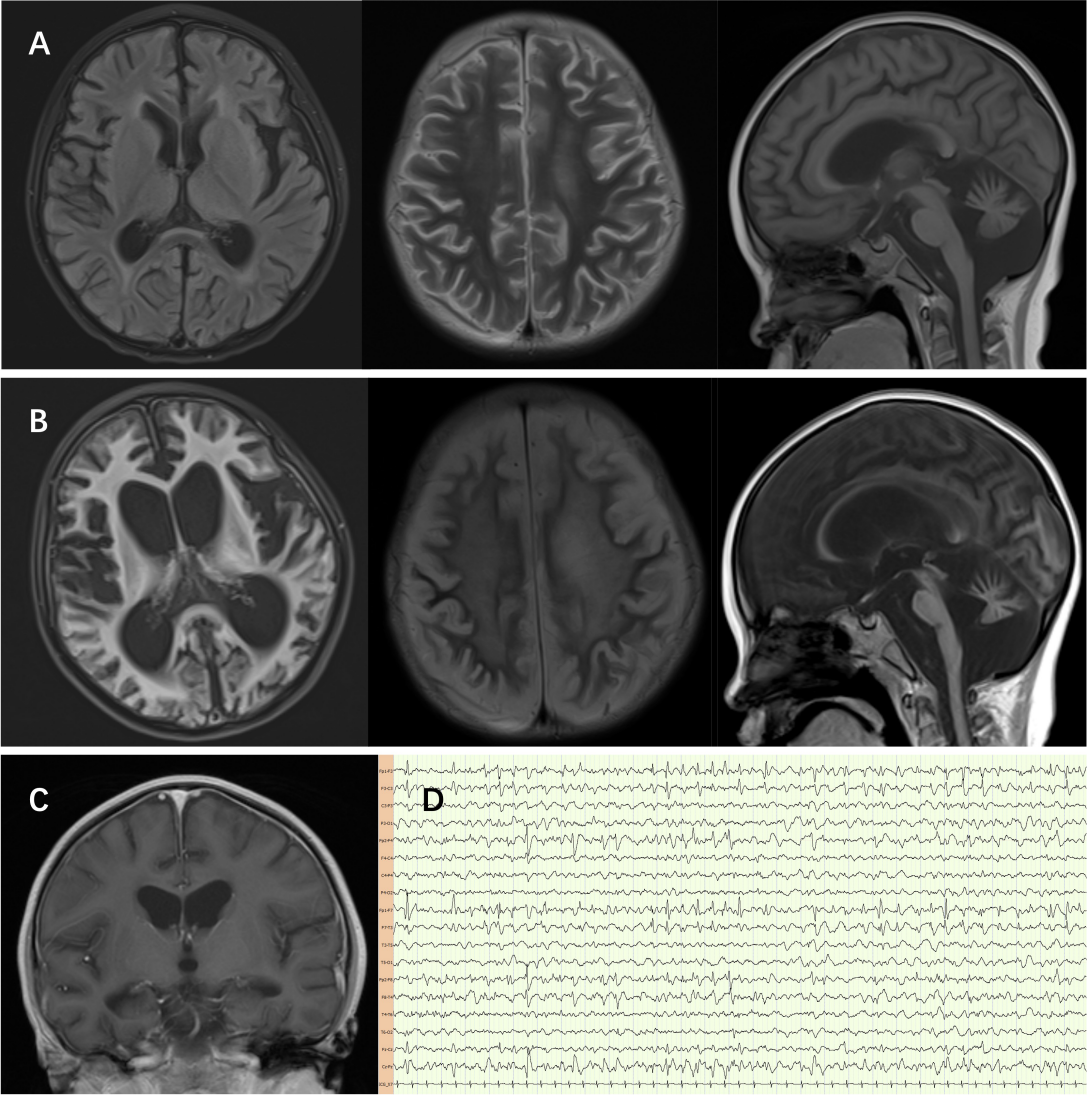
**(A)** The sulci of both cerebral hemispheres and cerebellar hemispheres are deepened, the gyri are thin, the white matter is reduced, the extracerebral space is widened, the ventricles are full, the cisterna magna is widened, and patchy FLAIR slightly high signals can be seen in the frontal, temporal, insular lobes and the anterior and posterior horns of the lateral ventricles. The FLAIR signal along the sulci and gyri of the cerebellum is slightly high, and the T2 and FLAIR signals of the right thalamus are slightly high. **(B, C)** Diffuse atrophy of the cerebrum and cerebellum worsened after 1 year follow-up. **(D)** Electroencephalography revealed interictal bilateral frontal and temporal spike-and-wave discharges, no periodic discharges nor epileptic seizures.

Cerebrospinal fluid (CSF) examination at 5.5-year-old showed leukocytes of 21 x 10^6^/L with 89% mononuclear cells, protein of 1054 mg/L, glucose of 2.81 mmol/L, and negative next-generation sequencing (NGS) for pathogens. Serum and CSF were negative for antibodies to measles, influenza A and B, adenovirus, coxsackievirus, echovirus, Epstein-Barr virus, and herpes simplex virus. Magnetic resonance imaging at 5.5-year-old showed atrophy-like changes in the brain, full ventricles with periventricular edema, and the posterior lobe of the pituitary gland shows a slightly blurred and reduced high signal in the large cisterna magna, which worsened at follow-up 1 year later (**Figures 2A–C**). Electroencephalography revealed interictal bilateral frontal and temporal spike-and-wave discharges, no periodic discharges nor epileptic seizures (**Figure 2D**).

## Discussion

The patient’s clinical history began with recurrent infections in early infancy, and although immunological testing at 3 months of age revealed severe hypogammaglobulinemia and B-cell deficiency, further diagnostic work-up was lacking at that time, and the pathogenic *BTK* variant was not identified by genetic testing until 5 years of age, which delayed the definitive diagnosis of XLA and likely contributed to the progression of the neurodegeneration. This is consistent with previous reports that delayed diagnosis of XLA is associated with higher morbidity, including chronic lung disease and irreversible neurological damage (6). Early recognition of XLA, especially in male infants with recurrent bacterial infections, is crucial for timely initiation of IVIg therapy and reduction of complications, allowing children with XLA to survive into adulthood. In this case, the failure to reduce the frequency and severity of infections after treatment may be related to the early age of onset of the disease, but to late diagnosis and initiation of treatment. IVIg intervals of no more than every 28 days are recommended. Due to the limited medical resources and the economic reasons of the children’s family, we tried our best to ensure that the children applied it once a month, with the longest interval occasionally being 8 weeks.

Patients with XLA are susceptible to CNS infections and enteroviruses are the main source of infection (7–9). XLA enteroviral encephalitis presents with an insidious onset of slowly progressive loss of motor and cognitive abilities over a period of 2-3 years, followed by spastic quadriplegia, coma and death in almost 44% of cases (10, 11). The progressive neurodegeneration and CSF changes with mild lymphocytic inflammation in our child led us to suspect chronic active infections, including enterovirus, JC virus-associated leukoencephalopathy and subacute sclerosing panencephalitis (SSPE) due to measles virus infection. SSPE is a rare but devastating long-term complication of measles virus infection or (in rare cases) infection with live attenuated vaccine strains (12, 13). Measles antibodies were not detected in this case. It is difficult for XLA patients to produce neutralizing antibodies, and the negative antibody test results cannot exclude the possibility of measles virus infection. The difficulty in producing neutralizing antibodies against the primary cause of the disease prevented us from confirming our suspicion by serological testing. Meanwhile, negative PCR and NGS results cannot rule out infection, which makes the diagnosis of infection in patients with XLA very difficult, and the diagnosis in this case may need to be confirmed by biopsy of viral inclusion bodies from brain tissue. Long-term follow-up showed no significant reduction in the rate of intestinal infections in the patient after IVIg replacement therapy (14), the refusal of brain biopsy led to a delay in diagnosis, and there was a lack of effective antiviral therapy. Although adequate trough levels of IVIg replacement therapy can prevent bacterial infections, with one study suggesting a median dose of 397 mg/kg (15), it did not protect against intestinal viral infections.

XLA does not usually directly affect the nervous system. However, as early as 1996 (5), reported encephalomyelitis in 13 cases of primary immunodeficiency disease (PID), with 10 deaths, and enteroviral infections may have been important, as CSF from 7 patients was positive by polymerase chain reaction (PCR) or enterovirus culture. Ziegner et al. reported progressive neurodegeneration in 14 cases of PID who received long-term IVIg (16). Symptoms including loss of speech and memory and cognitive impairment, ataxia and incoordination, seizures and tremors, spasticity and weakness, electroencephalography with slow waves and/or epileptiform discharges, CSF may have elevated lymphocytes and proteins, and cranial imaging in all patients showed cerebral atrophy, whereas serum and CSF cultures of pathogens and PCR tests were inconclusive. Four of the patients were XLA, and the interval between the start of IVIg therapy and the development of neurological symptoms ranged from 3 to 13 years, and three died, with the interval between the first neurological symptom and death ranging from 1 to 9 years. Ziegner et al. hypothesized that cerebral atrophy was a complication of autoimmune reactions against brain tissue and IVIg therapy. In 2004, Shiroma et al. reported a case of XLA in a child aged 5 years with mental deterioration and gait disturbance, and a brain biopsy showed CD8-positive T-cell infiltration with cortical damage, suggesting that CD8 T-cell-mediated autoimmunity may have contributed to this serious complication (17). Not only infection manifestation but autoimmune symptoms may occur in patients with primary immunodeficiencies as well (18). Antibody-deficient autoimmunity is usually caused by dysregulated immune responses. Chronic inflammation resulting from subclinical infections is an important contributor to immune dysregulation in patients with XLA, even in the absence of B cells (19, 20). The autoimmune manifestations of XLA include inflammatory bowel disease (21, 22), chronic immune thrombocytopenia (23, 24), autoimmune renal disorders (25, 26) and Shulman disease (27). Our child’s neurological symptoms appeared almost 5 years after the onset of the disease (first infection) and before IVIg treatment, and we considered the presence of an autoimmune response as a mechanism for their occurrence. Interestingly, he showed elevated CD4^+^ T cells and naive CD4^+^ subsets, which may reflect compensatory immune activation due to chronic infection. However, the Th1/Th2/Th17/Treg subsets were normal, suggesting that T-cell dysregulation is not a major driver of disease progression.

Given this case’s tragic outcome, and the failure of standard IVIg to prevent CNS complications, several interventions could be considered for similar patients. First, in patients with XLA/PID and signs of CNS involvement, routine CSF virological and autoimmune testing is necessary, the examination of cerebrospinal fluid should be expanded to include flow cytometry evaluation. Then, high-dose IVIG, intrathecal immunoglobulin, or experimental antivirals (e.g., ribavirin for enterovirus) could be tried. Although live vaccination is a contraindication to XLA, the theoretical risk of vaccine-associated SSPE in immunodeficient hosts warrants further study. SARS CoV2 infection could affected the course of the disease and the time to viral shedding was proportional to the symptomatic period and prolonged in children with XLA (28). As suggested by Hassin et al., (14) We suggest to monitor trough values of pathogen-specific antibody (e.g., against measles, pneumococcus) may be helpful in tailoring IVIg regimens to ensure adequate protection, particularly in high-risk patients with previous CNS involvement.

## Conclusion

This case highlights the devastating consequences of the confluence of XLA, recurrent infections and neurodegenerative decline. Although chronic active enterovirus infection in the CNS may be the culprit, diagnosis is difficult and complex autoimmune mechanisms may be involved. Conventional IVIg may not provide adequate pathogen-specific antibody protection, and IVIg guided by antibody monitoring and using direct CNS administration may improve prognosis. Further research is needed to determine the optimal screening and treatment strategy for XLA-related neurological complications.

## Data Availability

The data generated in this study are not publicly available due to concerns regarding participant anonymity. Further inquiries should be directed to the corresponding authors.
